# Quantitative Digital Imaging of Banana Growth Suppression by Plant Parasitic Nematodes

**DOI:** 10.1371/journal.pone.0053355

**Published:** 2012-12-28

**Authors:** Hugh Roderick, Elvis Mbiru, Danny Coyne, Leena Tripathi, Howard J. Atkinson

**Affiliations:** 1 Centre for Plant Sciences, University of Leeds, Leeds, United Kingdom; 2 International Institute of Tropical Agriculture, Kampala, Uganda; Kansas State University, United States of America

## Abstract

A digital camera fitted with a hemispherical lens was used to generate canopy leaf area index (LAI) values for a banana (*Musa* spp.) field trial with the aim of establishing a method for monitoring stresses on tall crop plants. The trial in Uganda consisted of two cultivars susceptible to nematodes, a plantain, Gonja manjaya and an East African Highland banana, Mbwazirume, plus a nematode resistant dessert banana, Yangambi km5. A comparative approach included adding a mixed population of *Radopholus similis*, *Helicotylenchus multicinctus* and *Meloidogyne* spp. to the soil around half the plants of each cultivar prior to field planting. Measurements of LAI were made fortnightly from 106 days post-planting over two successive cropping cycles. The highest mean LAI during the first cycle for Gonja manjaya was suppressed to 74.8±3.5% by the addition of nematodes, while for Mbwazirume the values were reduced to 71.1±1.9%. During the second cycle these values were 69.2±2.2% and 72.2±2.7%, respectively. Reductions in LAI values were validated as due to the biotic stress by assessing nematode numbers in roots and the necrosis they caused at each of two harvests and the relationship is described. Yield losses, including a component due to toppled plants, were 35.3% and 55.3% for Gonja manjaya and 31.4% and 55.8% for Mbwazirume, at first and second harvests respectively. Yangambi km5 showed no decrease in LAI and yield in the presence of nematodes at both harvests. LAI estimated by hemispherical photography provided a rapid basis for detecting biotic growth checks by nematodes on bananas, and demonstrated the potential of the approach for studies of growth checks to other tall crop plants caused by biotic or abiotic stresses.

## Introduction

Establishing damage levels from biotic stresses and the resultant economic loss to crops requires optimisation between assuring the reliability of the information gained and the effort and resources invested in its acquisition. The plant parasitic nematodes that cause severe losses to global banana and plantain (*Musa* spp.) production provide a specific example of the difficulties of damage assessments. The yield losses they cause are a combination of reduced plant growth that extends the production cycle, reduced harvested banana bunch weight and toppling of plants with damaged root systems in storms [1], [2]. They are often controlled in commercial plantations by periodic application of pesticides [3] but chemical control is rarely affordable or available to small producers in African countries where losses may be up to 71±16% [4].

Damage and economic thresholds based on nematode densities recovered from roots have been determined for *Radopholus similis*, which is widely damaging in commercial plantations, but the values vary for different geographical regions [1], [5]. A second approach measures the extent of root necrosis caused by this nematode. The resultant disease index is readily obtained and can be related to economic thresholds [5]–[7]. However, this approach has limitations when additional economically damaging nematode species are present, as frequently occurs in Uganda [8]. In that country *Helicotylenchus multicinctus* and *Meloidogyne* spp. are also present in tropical lowland ecologies with *Pratylenchus goodeyi* present at cooler higher altitudes [8]. Work in Costa Rica on the dessert banana cultivar Grand Naine ranked *R. similis*, *P. coffeae*, *M. incognita* and *H. multicinctus* in descending order of severity of root damage by equal densities of each nematode species but recorded *M. incognita* as causing the largest reduction in bunch weight [9]. Similar work with the plantain cultivar Apantu-pa in Ghana identified that *P. coffeae* caused the most severe necrosis and yield reductions but both *M. javanica* and *H. multicinctus* caused losses of 30% and 26% respectively with relatively less necrosis [10]. Consequently root necrosis may not be a reliable basis for assessing nematode losses to yield when *Meloidogyne* spp. are one of the economic species present [11] or generally when *R. similis* is not the only damaging species present.

This work uses leaf area index (LAI) to measure banana plant growth checks by nematodes more directly than inferring them from nematode root density or damage. LAI is widely used to measure the growth of crop plants and can be defined as the amount of leaf surface area (single side) per-unit area of the ground. It relates to the capacity of the banana canopy to intercept solar radiation and fix carbon and varies with location, planting density and additional factors such as season [12]. LAI is important because leaves normally far outweigh the contributions from other plant parts to the yield of most crops [13]. A range of methods have been used to measure LAI. Those applied to crops include direct measurement of the lengths and widths of leaves [14] and approaches that use ground based instruments that measure the fraction of transmitted radiation that passes through a plant canopy [15],[16]. The approach taken in this work is based on hemispherical photography using a camera with a 180° fisheye lens directed from the ground to the sky zenith to capture an image. This is then analysed to determine the LAI for those plants in the field of view. Hemispherical photography has been shown to be an effective tool for estimating LAI in various settings, including for coniferous forests [17] and single urban trees [18]. The method has also been validated against other indirect methods for monitoring LAI [18]–[20]. The approach has benefited from the development of digital photography, avoiding the need to develop film camera images. Proprietary software for a personal computer rapidly processes digital images to LAI estimates.

The current study explores the use of digital photography and LAI to detect growth checks to banana plants using a replicated field trial design in Uganda imposed by challenge with a combination of *R. similis*, *H. multicinctus* and *Meloidogyne* spp. Two nematode susceptible banana cultivars were used together with Yangambi km5 that has known resistance against *R. similis* [21]. The differences detected that are attributable to nematode challenge were related to bunch weights and root necrosis over two successive cropping cycles, to provide a comprehensive assessment of the technique. The results establish the potential of the approach for studies of growth checks to banana and other tall crop plants including those caused by biotic or abiotic stresses.

## Methods

### Study Site

The trial was conducted at Sendusu (328340E, 08320N), 1150 m above sea level, 28 km north of Kampala, Uganda. The site receives mean annual rainfall of 1300 mm in a bimodal pattern and annual minimum and maximum mean temperatures of 16°C and 28°C. The trial site land had been fallow for one year following cultivation of yam and cassava. No endangered or protected species were involved in the described field study and no specific permits were required for the studies, which were carried out on land designated for field trials at the research station of the International Institute for Tropical Agriculture (IITA).

### Experimental Design

The trial included two factors: banana cultivar and nematode treatment. Three cultivars were used, the East African highland banana (EAHB) Mbwazirume (AAA-EA genotype), the plantain Gonja manjaya (AAB genotype) and the dessert banana Yangambi km5 (AAA genotype) which were selected as a representatives of different banana genotypes and nematode host status. All planting material was sourced from farms in close proximity to the Sendusu site. Suckers were pared and hot water treated for 30 sec at 98°C for disinfection of plant parasitic nematodes and weevil larvae [22], then potted into steam-sterilised sandy loam soil in 10 litre polythene bags. Nematode treatment comprised either no supplementation of the natural population or addition of approximately 5,500 nematodes to soil around plants prior to planting. Nematodes were added using 20 g of fresh banana root segments containing 77% *R. similis*, 17% *H. multicinctus* and 6% *Meloidogyne* spp. The inoculum roots were collected from an infested banana site at Sendusu and placed into a shallow excavation around each plant and mixed into the soil. Estimated nematode densities were calculated from five 5 g root segment sub-samples immediately prior to inoculation. Root pieces were bulked, chopped (∼0.5 cm) and sub-samples removed, macerated in a blender for 10 s and the nematodes extracted overnight using a modified Baermann technique [23]. Each plant not given added nematodes received 20 g of autoclaved root segments. All plants were planted into the field twenty days later on 18th May 2009. A standard split-plot design [24] was used with plot replicates of 36 banana plants spaced 3 m apart in a 6 by 6 grid. Each cultivar was replicated in eight plots, four of which were infected with nematodes giving a total of 144 plants for each cultivar/treatment combination. The cultivation followed local practises with 10 kg of dry cow manure added per planting hole and fresh elephant grass (*Pennisetum purpureum*) mulched four times annually around each plant. Water was applied daily for 1 week to ensure transplanted plants established. The trial was surrounded by a row of guard plants transplanted with the trialled plants.

### Crop Data Collection

Plant growth was assessed from 106 days post planting (dpp) every 14 days between 10 am and noon by hemispherical photography. Images were captured with an 8 mm full circle fisheye lens (EX-DG, Sigma, Kanagawa, Japan) on a full-frame digital camera (EOS 5D, Canon, Tokyo, Japan) attached to a self levelling mount (SLM7, Delta-T, Cambridge, UK) and a monopod. The camera lens front was placed 95 cm above the ground in the centre of four plants and directed at the sky zenith. A single exposure was taken while the observer knelt below the field of view. The majority of the canopy recorded in each image was dominated by the four plants surrounding the camera. Nine images per plot were taken, so that each plant was dominant in only one image. Mature bunch weight and date of harvest was recorded for each plant which spanned 350–520 dpp and 530–900 dpp for the first and second harvests respectively.

The crop was continually monitored by field observation and during analysis of hemispherical images for the foliar diseases Black Sigatoka, Fusarium wilt and bacterial *Xanthomonas* wilt and for the presence of the banana weevil *Cosmopolites sordidus.*

### Analysis of Hemispherical Photographic Images

The images collected by digital camera were imported into proprietary software (HemiView Version 2, Delta-T, Cambridge, UK) calibrated to the lens used from pre-loaded settings. The software classifies images to distinguish visible and obscured sky directions and so define canopy openings relative to banana foliage. The sky visibility and obstruction is then defined as a function of sky direction and canopy indices are calculated from this information to provide results including LAI [25]. The algorithms estimate LAI as half of the total leaf area per unit ground area by means of an iterative inversion of Beer’s Law [12]. LAI generated for each image was exported into Excel (Microsoft, Redmond, WA) before further statistical analysis. The mean of the measurements across four plots, 36 images in total, was calculated for each cultivar/treatment combination on each day of measurement.

### Nematode Damage Assessment

At harvest, the percentage root necrosis for each plant was derived from five randomly selected functional primary roots. They were cut into 10 cm lengths, sliced length-wise and the proportion of cortex necrosis estimated for each root piece [11]. The overall percentage root necrosis comprised the average of the five pieces. The five functional root segments used to score for root necrosis were cut into 0.5 cm sections, thoroughly mixed, and a 5 g sub-sample used for nematode extraction. Each sub-sample was macerated in a blender for 10 s and the nematodes extracted overnight as above. Nematode population densities were estimated from three 2 ml aliquots from a 25 ml suspension. Nematodes were identified to species level and population densities estimated per 100 g root fresh weight. At the first harvest nematode counts were made for all plants, at the second harvest nematode counts were made on three samples per plot. Nematode densities were log transformed before analysis.

### Statistical Analysis

All data were analysed using a standard statistical package (SPSS v18; IBM Corporation Armonk, New York, USA; http://www-01.ibm.com/software/analytics/spss/) installed on a personal computer with choice of method informed by a standard text [26].

## Results

### The Leaf Area Index of Growing Plants

**Figure 1:**
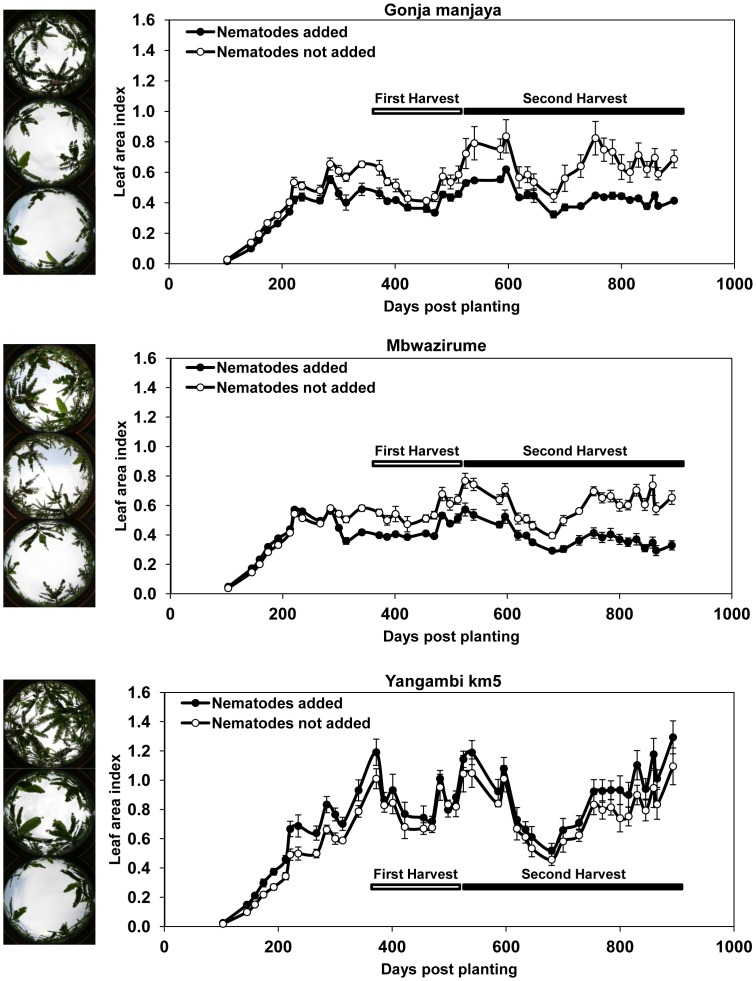
Leaf area index (LAI) measured by hemispherical digital photography. Mean canopy LAI measurements of four replicate plots of 36 banana plants per treatment of the susceptible cultivars Gonja manjaya and Mbwazirume and the resistant cultivar Yangambi km5 across two growth cycles with or without nematode addition of a mixed population of 5,500 nematodes per plant before transfer to the field. Error bars indicate ± SEM (n = 36) and the first and second harvest periods are indicated by open and closed bars, respectively. Adjacent images indicate example LAI values at the minimum, median and maximum values for each banana cultivar.

Figure 1 provides mean canopy LAI values based on 36 images for each data point. Images were collected over two growth cycles for plants with or without nematode addition before their transfer to the field. The images in the figure indicate the range of LAI values that were obtained. In the first cropping cycle, the highest grand mean value for LAI occurred at 340 dpp for Gonja manjaya and Mbwazirume. The mean (± SEM) for the images on this day for plants receiving nematodes before transplanting was 74.8±3.52% for Gonja manjaya and 71.1±1.88% for Mbwazirume of LAI values of 0.652±0.0154 and 0.587±0.0148, respectively, of plants not challenged by added nematodes. The highest grand mean LAI for the same two cultivars before the second harvest occurred at 540 dpp. When nematodes had been added to plants before transplanting, the mean values were 69.2±2.15% for Gonja manjaya and 72.2±2.74% for Mbwazirume of mean LAI values of 0.791±0.0406 and 0.743±0.0240, respectively, when no nematodes were added. A small opposite effect of adding nematodes was observed for the nematode resistant Yangambi km5 plants. Mean values at the same dpp as used for the other two cultivars were 118.3%±5.52% and 113.4±4.16% for the first and second crop cycles relative to LAI values when nematodes were not added of 0.787±0.0307 and 1.048±0.0471 respectively. The difference in LAI between adding and not adding nematodes was statistically significant for all three cultivars (P<0.001, for both occasions of Gonja and Mbwazirume and P<0.05 for both Yangambi km5 comparisons; n = 36 for each mean, Oneway Anova with *apriori* contrasts).

### Nematode Densities

**Figure 2:**
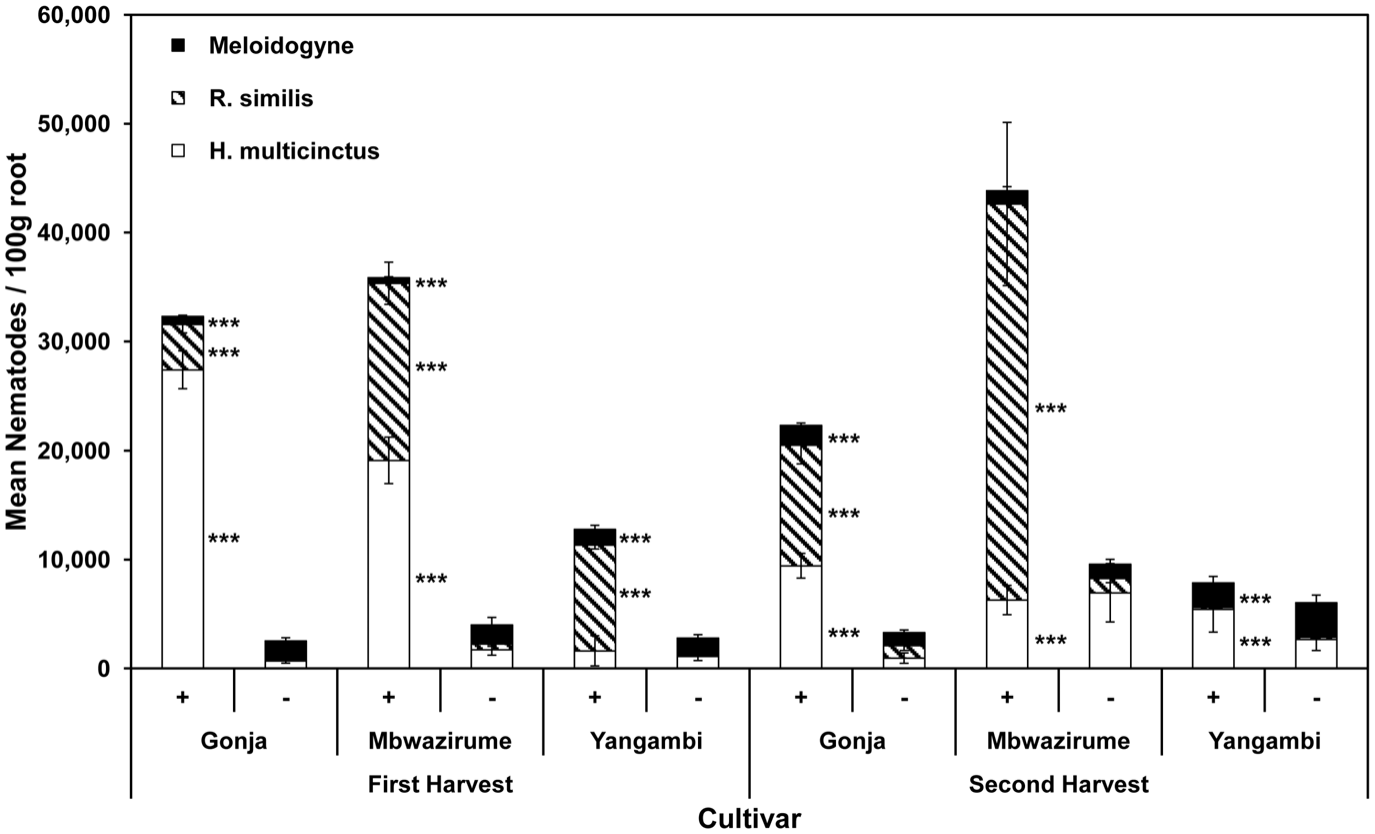
Nematodes recovered at harvest. Mean numbers per 100 g root of each of the three added nematode species recovered at first and second harvest on each of the three banana cultivars. Error bars indicate ± SEM (Harvest 1 n = 144; Harvest 2 n = 12). Univariate analysis was used to detect differences between nematodes added (+) and not added (–) treatments (***–P<0.001).

For the two susceptible cultivars, the addition of nematodes increased the number of *R. similis* and *H. multicinctus* and decreased the number of *Meloidogyne* spp. recovered at both harvests relative to those plants not receiving that treatment. A low level of invasion occurred in plants not receiving a nematode treatment prior to transfer to the field from the nematode population in the field soil at the site (Figure 2). The density of *R. similis* relative to *H. multicinctus* increased from the first to second harvests on both susceptible cultivars. Nematode addition also led to a higher density of nematodes recovered from the resistant cultivar, Yangambi km5, at both harvests compared to the plants not receiving additional nematodes, but less than was seen on the susceptible cultivars. The values are statistically significant (P<0.001) with the exception of the non-abundant *H. multicinctus* on Yangambi km5 at the first harvest and *Meloidogyne* spp. on Mbwazirume and *R. similis* on Yangambi km5 at the second harvest.

### Leaf Area Index and Nematode Densities

**Figure 3:**
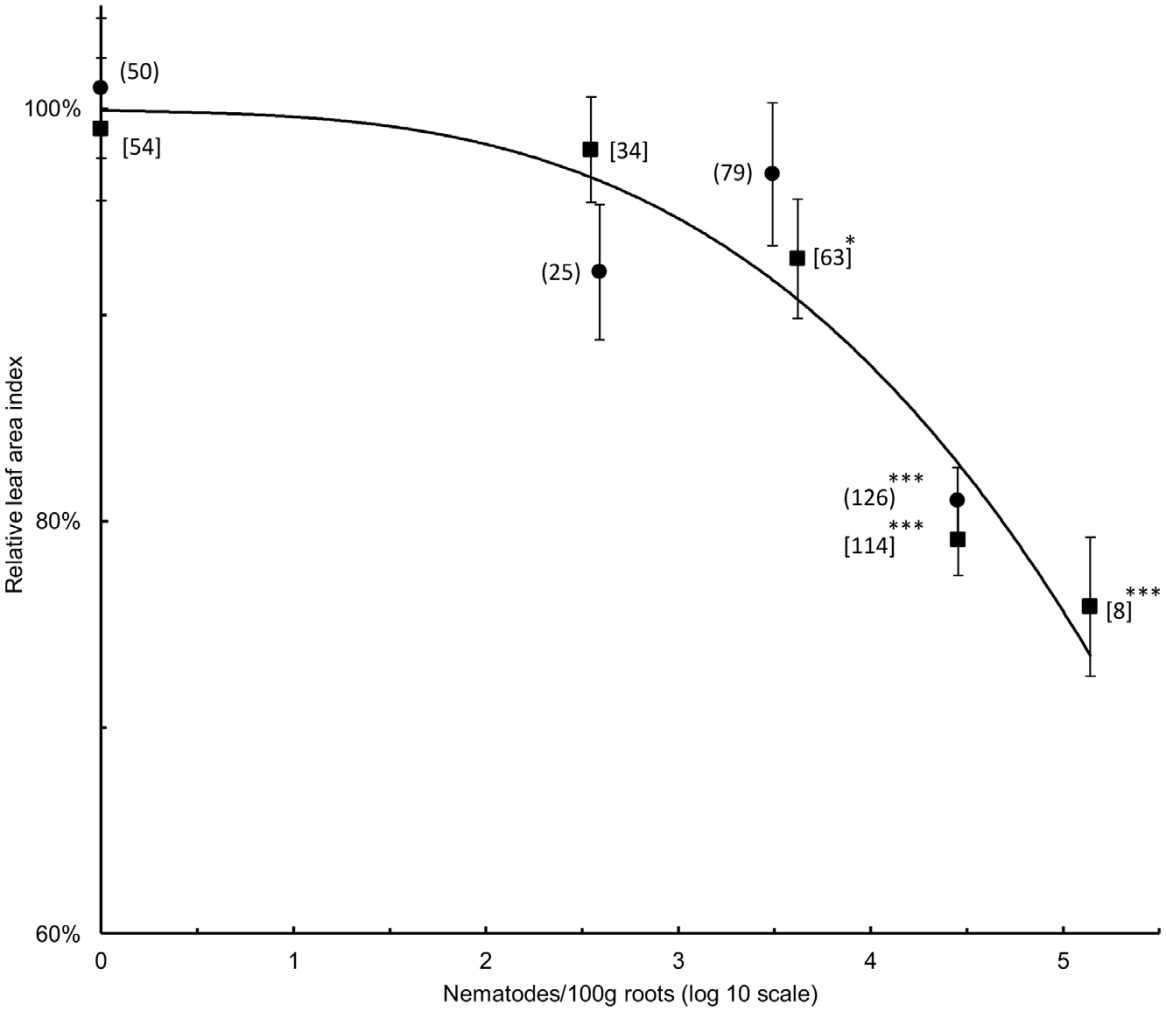
Relationship between LAI and nematode density. A cubic regression curve is fitted for the relationship for the cultivars Gonja (•) and Mbwazirume (▪) between LAI and mean nematode density for values within one of up to five nematode density classes (0; 1–<1,000; 1,000–<10,000; 10,000–<100,000 and 100,000 or more/100 g roots) for two harvests for both plants to which nematodes were and were not added prior to planting. All nematode density classes for which there is significant percentage reduction in LAI relative to when no nematodes were recovered from roots is shown for both cultivars (*–P<0.05; ***–P<0.001, Oneway Anova with *apriori* contrasts). The values in parenthesis are the number of values associated with each mean; (n), Gonja and [n] Mbwazirume.

The relationship between LAI and nematode densities was described by a cubic regression curve fitted to LAI values and nematode densities at harvest (Figure 3). There were similar decline curves for LAI values with increasing nematode density at both harvests for cultivars Gonja and Mbwazirume (P<0.001 in both cases; not shown). The data were pooled for the two cultivars and summarised with a cubic regression using mean LAI values at logarithmic mean nematode densities for a range of density classes. The first significant fall in LAI to 92.7±2.89% of 0.565±0.0111 in the absence of recovered nematodes occurred at a mean density of 4,166±348 nematodes/100 g roots for Mbwazirume (P<0.05; Figure 3). A significant decline in LAI for Gonja was established at the next higher density class with a mean LAI of 80.0±1.58% of 0.504±0.0172 at a mean density of 28,248±970 nematodes/100 g roots (P<0.001; Figure 3). The resistant cultivar Yangambi km5 showed no significant decrease from a LAI of 0.840±0.0307 as the nematode density increased and so this cultivar is not included in Figure 3.

### The Influence of Nematodes on Banana Bunch Weight

**Figure 4:**
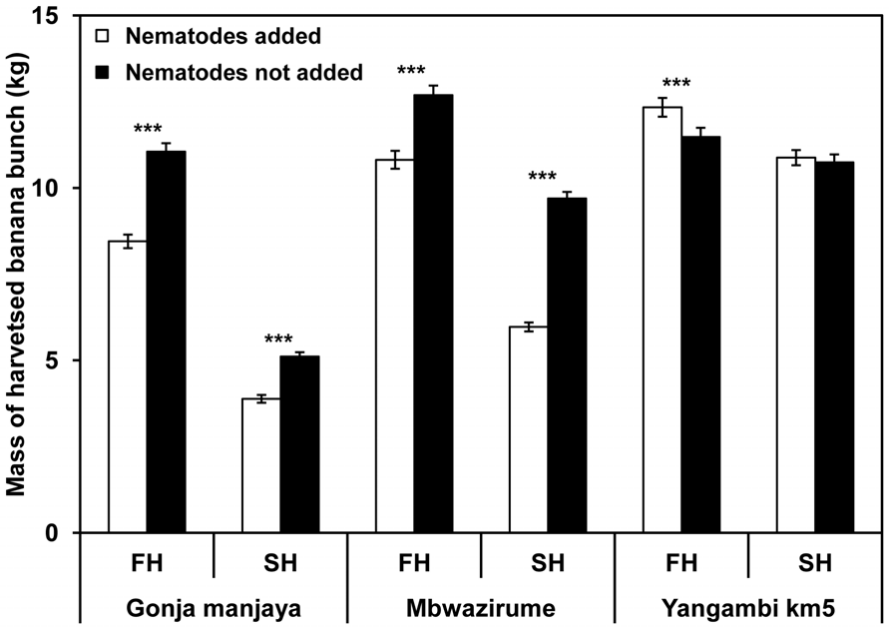
Harvested banana bunch weights. Mean values for each treatment for the three banana cultivars at first and second harvest. Error bars indicate ± SEM (Harvest 1 n≥120; Harvest 2 n≥95). Univariate analysis was used to detect significant differences (***–P<0.001) between bunch weights of nematodes added and not added treatments at first harvest (FH) and second harvest (SH).

Bunch weights were lower for Gonja manjaya and Mbwazirume for both harvests (univariate ANOVA) on plants receiving added nematodes compared to plants that did not (Figure 4). Bunch weights were lower when nematodes had been added by 23.5±1.82% and 24.0±2.34% for Gonja manjaya and 14.8±2.13% and 38.0±1.42% for Mbwazirume, for the first and second harvests respectively. Yangambi km5 showed a slight increase in yield when nematodes had been added of 7.0±2.45% and 1.3±2.08% at the first and second harvests respectively.

### Necrosis of Roots

**Figure 5:**
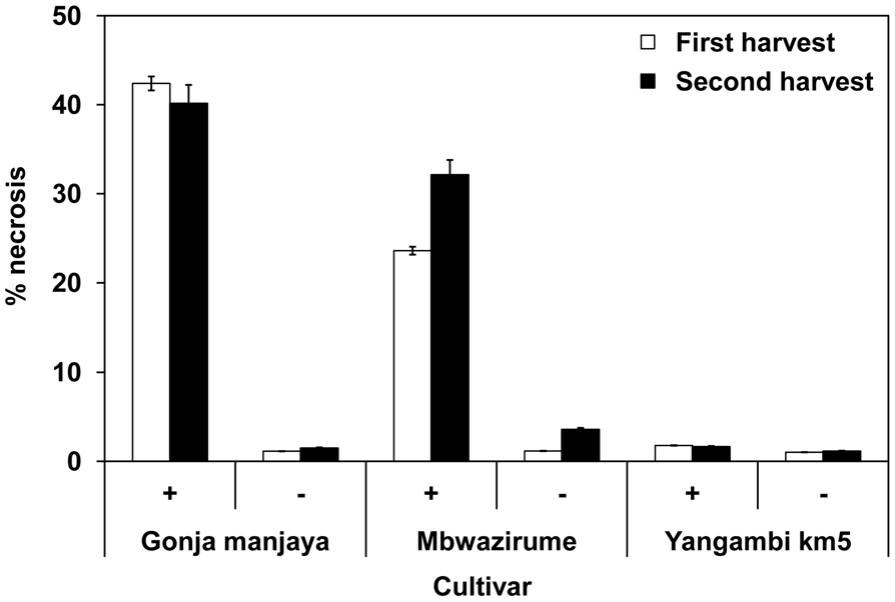
Percentage root necrosis at harvest. Mean values for each treatment for the three banana cultivars at first and second harvest. Values presented are back transformed from log necrosis values. Error bars indicate ±SEM (Harvest 1 n≥120; Harvest 2 n≥95)**.**

There was only limited necrosis observed on roots of plants not receiving nematodes before field planting and for Yangambi km5 roots with or without nematodes added (Figure 5). In contrast considerable necrosis was observed on both Gonja manjaya and Mbwazirume plants that had received nematodes. It was more severe on Gonja manjaya than Mbwazirume over both harvests (P<0.001; univariate analysis). Coefficients of the linear equation between necrosis and the nematode density were obtained by a stepwise entry of eligible variables. *R. similis* contributed most to the correlation coefficient (0.596), which increased to 0.628 when *H. multicinctus* was also entered with both additions being statistically significant (P<0.001). The partial correlation coefficient for each nematode for its linear effect on necrosis when the contribution of the other nematodes was removed was also statistically significant at 0.512 and 0.246 respectively (P<0.001 in both cases). The small contribution made by *Meloidogyne* spp. was not significant and so was excluded from the final model.

### The Incidence of Toppling

During the first cropping cycle 41 plants of Mbwazirume and Gonja manjaya plots to which nematodes had been added toppled. No plants toppled in plots without added nematodes for these two cultivars and no Yangambi km5 plants toppled irrespective of treatment (Table 1). In the second cropping cycle plants from all three cultivars toppled, mostly those to which nematodes had been added and less so for Yangambi km5, which had the same level for both treatments. Toppling incidence was similar when nematodes had been added for the two cropping cycles for Mbwazirume but increased significantly (P<0.01) for Gonja manjaya in the second cycle.

**Table 1:** The impact of the addition of nematodes prior to transplantation into the field on the number of standing (S) and toppled (T) banana plants during two crop cycles.

	First crop cycle						Second crop cycle					
	Nematodesadded		Nematodesnot added		χ^2^ P Value		Nematodes added		Nematodes not added		χ^2^ P Value	
Cultivar	S	T	S	T	addedvs not	*cf.*Mbw+Gonja	S	T	S	T	addedvs not	*cf.*Mbw+Gonja
Mbwazirume	120	24	144	0	<0.001	NS	115	29	140	4	<0.001	<0.01
Gonja manjaya	127	17	144	0	<0.001		95	49	136	8	<0.001	
Yangambi km5	144	0	144	0	NS		140	4	140	4	NS	
Total	391	41	432	0			350	82	416	16		

### Plant Height, Girth and Time to Harvest

A significant reduction in plant height at harvest was caused by adding nematodes before planting for both harvests with Gonja manjaya and Mbwazirume but the effect was only 5% and 12–15% for the first and second harvests respectively. The additional nematode presence also resulted in a 10–16% reduction in girth of Gonja manjaya at both harvests and Mbwazirume for the second harvest only. Similarly, there was a small, significant reduction in days to the first harvest for Mbwazirume and an increase for Yangambi km5 but no significant difference between treatments for Gonja manjaya (Table 2).

**Table 2:** The impact of the addition of nematodes prior to transplantation into the field on days to harvest, plant height at harvest and pseudostem girth at harvest for two crop cycles.

Cultivar	Treatment	Days to harvest		Plant height(cm)		Pseudostem girth (cm)	
		Firstharvest	Secondharvest	Firstharvest	Secondharvest	Firstharvest	Secondharvest
Gonja manjaya	Nematodes added	442.3 ±3.34	714.9 ±8.55	255.4 ±3.72**	218 ±4.69***	43.8 ±0.542***	37.2 ±0.850***
	Nematodes not added	440.3 ±3.05	712.8 ±6.72	269.4 ±2.28	257.6 ±4.77	48.4 ±0.389	44.5 ±0.824
Mbwazirume	Nematodes added	363.6 ±2.48 **	630.7 ±7.38	231.3 ±1.54***	231.2 ±2.93***	47.9 ±0.450	46.8 ±0.718***
	Nematodes not added	376.4 ±3.03	617.1 ±6.46	243.7 ±1.44	264.1 ±2.92	53.9 ±3.63	52.5 ±0.750
Yangambi km5	Nematodes added	411.3 ±3.12 ***	613.1 ±5.62*	198 ±1.81	250.7 ±3.38	38.1 ±0.453	43.5 ±0.801
	Nematodes not added	431.9 ±4.01	636 ±7.18	197.7 ±1.90	245.1 ±3.98	38.7 ±0.401	42.3 ±0.753

*** P<0.001;

** P<0.01;

* P<0.05; t-test comparison between the two treatments for each cultivar at harvest. Each mean (± SEM) is based on 83–138 and 27–137 plants for the first and second harvests, respectively.

### The Incidence of Other Biotic Stresses

Plants were examined in the field for foliar diseases on each day that hemispherical images were captured for LAI analysis and again during the analysis of the images. Initial symptoms of Bacterial wilt were noticed for only 15 of the 766 (2%) standing plants during the second crop cycle. These plants were immediately destroyed on detection of the disease. Black Sigatoka and Fusarium wilt were absent or present at such a low level that damage to leaves was not observed. Weevil damage was detected for just 5 plants but not until close to the second harvest. The low incidence of these biotic stresses is likely due to the deliberate sighting of the trial away from established banana plantations and the use of land that had been fallow for a year following cultivation of cassava and yam to ensure LAI differences detected could be attributed to nematodes.

## Discussion

Measurement of LAI by hemispherical digital photography provided a rapid method of assessing banana growth. The approach provides a basis for the continual measurement of a wide range of stresses on the growth of tall crops such as banana under field conditions. Its potential was established in this work using the example of nematode challenge, which is a known major biotic stress for banana crops. The blocked and replicated field trial design ensured variation in growth between plants in the same treatment did not prevent the effects under study reaching statistical significance. The design enabled the decrease in LAI values recorded with high nematode density to be related to the biotic stress the animals imposed. It incorporated a comparative approach which revealed a lower LAI for those plants with a substantially higher nematode density at harvest after their addition prior to planting relative to those plants challenged only by the lower natural population in the soil. In addition, the nematode resistant cultivar Yangambi km5 was included and it showed no decrease in LAI with high nematode density in contrast to the two susceptible cultivars Mbwazirume and Gonja manjaya. There was also only a low incidence of the other major biotic stresses in Uganda, bacterial *Xanthomonas* wilt, Fusarium wilt, Black Sigatoka and weevils, such that their contribution to mean and variability of the LAI values was limited.

The LAI values increased as the plants grew in an asymptotic manner to a plateau phase over the first cropping cycle with the increasing leaf area being completed by 340 dpp for both Mbwazirume and Gonja manjaya. Yangambi km5 grew vigorously and its LAI values reached a higher value than the other two cultivars. The plant growth pattern measured by LAI was less evident for the second crop of the cultivars as second generation plants had grown considerably before the first harvest removed the contribution of mother plants to the values. The LAI also fluctuated with the impact of climatic factors such as storms. The addition of nematodes to soil around roots before planting out to the field reduced the subsequent LAI for both Mbwazirume and Gonja manjaya from about 300 dpp relative to those not receiving this treatment. The difference in the LAI for these cultivars between the two treatments increased across the two cropping cycles, indicating that a progressive suppression of growth was occurring. Yangambi km5 showed a slightly increased LAI in the presence of added nematodes over both cropping cycles, indicating that in this study this cultivar responds to nematode infection with stimulated growth. Such an effect has previously been reported under certain circumstances such as low nematode densities [27]. The suppression of peak LAI by 25–31% for Mbwazirume and Gonja manjaya, when nematodes were added before planting, was greater than the change of 5–11% found in plant height at harvest. Pseudostem girth has been used to predict yields [14] but no significant reduction was recorded at first harvest of Mbwazirume in this work, although its yield was reduced significantly by the added nematode challenge. The results suggest LAI is superior to both these other measures of plant size for detecting the impact of nematodes on banana plant growth.

The LAI values are lower than recorded previously in other parts of the world but similar to previous values for EAHB obtained by measuring individual leaf dimensions at the same locality as this trial [14]. This relatively low value is partly because the trial planting density was typical of plantations in Uganda which are less than in many commercial plantations elsewhere. Presumably they are optimal for EAHB based on grower experience given a cultivation history in East Africa of at least 1,000 years [14].

The relationship between growth suppression, as measured by LAI, and nematode numbers fitted a polynomial curve (Figure 3) as used previously to relate nematode density to root necrosis of banana [7], [9] and conformed in shape to that derivable on a theoretical basis [28]. It was validated by comparing LAI values at different levels of nematode challenge and by measurement of root necrosis at harvest. The addition of nematodes resulted in considerable necrosis of the root systems of Gonja manjaya and Mbwazirume but not Yangambi km5 at both harvests. The nematodes were responsible for initiating much of the necrosis as it was much less evident when they were not added to the soil around plants. Correlations from linear regression indicate *R. similis* contributes more to necrosis than *H. multicinctus* with *Meloidogyne* not contributing significantly to this effect. The necrosis associated with *R. similis* causes structural damage to roots leading to an incidence of toppling [29]. In the current study, necrosis of Mbwazirume roots increased proportionally with *R. similis* densities over the two cropping cycles. However, considerable necrosis was associated with more *H. multicinctus* than *R. similis* in roots of Gonja manjaya by first harvest. This damage did not become more severe by the second harvest although an increase in the density of *R. similis* was recorded. This may indicate that the *H. multicinctus* population caused much of the necrosis of Gonja manjaya roots and indicate variability in responses of different cultivars to complex populations of parasitic nematodes.

The nematode density recovered from Gonja manjaya at harvest of the second crop cycle was also less than the first. This effect may relate to the influence on nematode densities of the root-carrying capacity of the damaged root system as a crop grows, as studied for *Pratylenchus zeae* on rice [30]. Possibly the high density of nematodes present towards the end of the first cropping cycle of this plantain suppressed early root growth in the second cycle so reducing the subsequent root-carrying capacity for nematodes. In contrast, Mbwazirume had greater nematode densities associated with its roots at harvest of the second cycle than the first. It may have offered a larger root system than Gonja manjaya, which consequently reduced toppling incidence and allowed a subsequent build up of the nematode population to levels that resulted in a higher loss of yield by the second harvest.

It has been suggested that in Uganda a disease index in excess of 10, the equivalent of 273 *R. similis*/100 g root, can result in economic damage at an altitude of 1200 m above sea level [5]. This is a considerably lower nematode density than obtained in the current work at harvest even when no nematodes were added and limited necrosis occurred. Disease indices based on root necrosis alone may be an unreliable predictor of economic damage, particularly in the complex situations such as occurs in Uganda. A combination of percentage dead roots, number of large lesions and the nematode population density was used to measure *Musa* genotype field response to *R. similis* and *H. multicinctus* [31]. That approach is labour intensive and requires more specialist expertise than required to measure LAI as in the current study. In our work we selected three distinct banana genotypes to optimise the outcome but the comparison of planting types needs to be expanded. For instance, there is a considerable range of EAHB cultivars with five distinct genotypes [14] that may have differing responses to nematode presence in roots.

We have shown that LAI provides a continual measure of the impact of a plant stress on banana growth at a locality for particular genotypes but like other measurements of crop growth and yield in the field it is an integrative measure. As such studies need to be designed so that it can be demonstrated that the stress considered is the major factor in the outcome recorded. Therefore the cause of the effect on growth, such as the density of nematodes, should be confirmed to define the one or more plant stresses principally involved. This is, however, a requirement of all methods of assessing nematode induced losses to banana in field conditions as none provide measures that are independent of the consequences of other factors and stresses. For instance the level of necrosis is sometimes not reflected in yield loss in the absence of banana toppling for both *R. similis* in the Cameroon [32] and *P. goodeyi* in Rwanda [33]. In otherwise favourable conditions, the sum of all stresses on these plants may not check growth. The proportion of root mass undamaged by nematodes may be sufficient for normal physiological functions but inadequate to prevent the plant from toppling during storms and high winds. More work may establish that LAI at a specific crop growth stage offers a reliable approach for defining damage thresholds for banana crops that can be related to even complex stresses, such as a combination of nematode species.

Although the yield loss contributions due to toppling and bunch weight loss of standing plants differed for Gonja manjaya and Mbwazirume the overall values were similar. The combined yield loss including toppled plants on plots receiving nematodes over the second cropping cycle was significantly larger for Gonja manjaya and Mbwazirume than the losses over the first cycle. This demonstrates progressive and severe nematode induced losses in perennial banana plantations of susceptible cultivars that can be inflicted within the first few crop cycles. Loss estimates in excess of 50% have been reported from several studies [4] and emphasises the need for improved nematode control of this important food security crop in sub-Saharan Africa.

The results establish that LAI has potential for continual measurement of stress on the growth of tall crops, such as banana under field conditions. We will next apply this approach in a contained field trial to assess yield responses to nematode challenge of recently developed transgenic plantains with resistance to *R. similis* and *H. multicinctus* [34]. This will establish whether LAI is a reliable diagnostic tool for assessing nematode resistance in such plants.
